# Draft Genome Sequence of *Janthinobacterium* sp. Ant5-2-1, Isolated from Proglacial Lake Podprudnoye in the Schirmacher Oasis of East Antarctica

**DOI:** 10.1128/genomeA.01600-15

**Published:** 2016-01-21

**Authors:** Hyunmin Koo, Bailey M. Strope, Eddy H. Kim, Adel M. Shabani, Ranjit Kumar, Michael R. Crowley, Dale T. Andersen, Asim K. Bej

**Affiliations:** a Department of Biology, University of Alabama at Birmingham, Birmingham, Alabama, USA; b CCTS Biomedical Informatics, University of Alabama at Birmingham, Birmingham, Alabama, USA; c Heflin Center for Genomic Sciences, University of Alabama at Birmingham, Birmingham, Alabama, USA; d Carl Sagan Center, SETI Institute, Mountain View, California, USA

## Abstract

*Janthinobacterium* sp. Ant5-2-1, isolated from the Schirmacher Oasis of East Antarctica, produces a purple-violet pigment, manifests diverse energy metabolism abilities, and tolerates cold, ultraviolet radiation, and other environmental stressors. We report here the 6.19-Mb draft genome of strain Ant5-2-1, which will help understand its survival mechanisms in extreme Antarctic ecosystems.

## GENOME ANNOUNCEMENT

*Janthinobacterium* sp. Ant5-2-1, isolated from the seasonally frozen Proglacial Lake Podprudnoye in the Schirmacher Oasis of East Antarctica, is a psychrotolerant Gram-negative bacillus that thrives in extreme cold, dry, and high solar ultraviolet (UV) radiation environments (1). Previously, it has been reported that a violacein-like purple-violet pigment (PVP) produced by Ant5-2 absorbs UVA, UVB, and UVC radiation and manifests strong antimicrobial and anticancer activity (1–5). We describe here a draft genome of *Janthinobacterium* sp. Ant5-2-1 to investigate the key metabolic, stress-responsive, and pigment-producing genes necessary for their survival and sustenance in extreme Antarctic conditions.

Purified genomic DNA from an Ant5-2-1 culture was sequenced on an Illumina MiSeq (250-bp, paired-end), generating 2,389,439 reads. The adapter sequences were checked by FastQC (6) and then low-quality regions were trimmed by Trimmomatic (7) and Cutadapt (8). Optimal assembly parameters were determined through VelvetOptimiser (9), and then Velvet was used with a *k*-mer length of 167 for *de novo* assembly (10). The final draft assembly resulted in 179 contigs with 62.5% GC content and a total length of 6,196,351 bp with 290× coverage. The contig sizes were from 502 to 286,011 bp, with a mean length of 34,616 bp and an *N*_50_ of 92,795.

The assembled genome was annotated using the Rapid Annotations using Subsystem Technology (RAST) server (11). The result showed 5,536 protein-coding genes (CDSs), including 2,553 known and 2,983 unknown subsystems. Using tRNAscan-SE (12), RNAmmer (13), and ARAGORN (14), we detected 7 rRNAs, 80 tRNAs, and 1 tmRNA.

We found various energy metabolism genes: 80 sulfur, 48 phosphorus, 178 carbon, and 56 nitrogen (nitrate and nitrite ammonification, ammonia assimilation, and nitrosative stress) categories. Additionally, we identified 138 cell wall and capsule genes, including 31 genes for the biosynthesis of capsular and extracellular polysaccharides; 2 quorum-sensing genes (*qseB* and *qseC*); 228 flagellar motility and chemotaxis genes; 116 antibiotic resistance–conferring genes (fluoroquinolones, fosfomycin, beta-lactamase, and the MATE family of multidrug-resistance efflux pumps); and 58 iron acquisition and metabolism genes (hemin transport system). In the environmental stress management category, we found 90 genes related to DNA repair (*recA*, *recX*, UvrABC system, *uvrD*); 8 genes for programmed cell death and toxin-antitoxin systems (bacterial caspases, murein hydrolase regulation, and cell death); and 200 genes for stress management (osmotic stress, oxidative stress, protection from reactive oxygen species, cold shock, detoxification, carbon starvation, and sigmaB stress response regulation). Additionally, we identified genes for tryptophan and PVP biosynthesis pathways. Finally, secondary metabolites were found using antiSMASH (15), giving gene clusters (terpene, bacteriocin, and PVP pigment biosynthesis genes).

Diverse energy metabolism, quorum sensing, antimicrobial resistance, stress-responsive genes, along with exopolysaccharide and pigment biosynthesis pathways in the genome of Ant5-2-1 will allow us to better understand the adaptation mechanisms manifested by this bacterium in extreme Antarctic environments, and its importance in biomedicine and biotechnology.

### Nucleotide sequence accession numbers. 

This whole-genome shotgun project has been deposited in DDBJ/ENA/GenBank under the accession number LNCE00000000. The version described in this paper is the first version, LNCE01000000.
